# Accuracy and reliability of the InBody 270 multi-frequency body composition analyser in 10-12-year-old children

**DOI:** 10.1371/journal.pone.0247362

**Published:** 2021-03-26

**Authors:** Malte Nejst Larsen, Peter Krustrup, Susana Cristina Araújo Póvoas, Carlo Castagna

**Affiliations:** 1 Department of Sports Science and Clinical Biomechanics, SDU Sport and Health Sciences Cluster (SHSC), Faculty of Health Sciences, University of Southern Denmark, Odense, Denmark; 2 Department of Sport and Health Sciences, College of Life and Environmental Sciences, University of Exeter, Exeter, United Kingdom; 3 Department of Physical Education and Sports Training, Shanghai University of Sport, Shanghai (SUS), China; 4 Research Center in Sports Sciences, Health Sciences and Human Development, CIDESD, University Institute of Maia, ISMAI, Maia, Portugal; 5 Technical Department, Fitness Training and Biomechanics Laboratory, Italian Football Federation, Coverciano, Florence, Italy; 6 School of Sport and Exercise Sciences, University of Rome Tor Vergata, Rome, Italy; University of Cádiz, SPAIN

## Abstract

The aim of this study was at examining the validity and reliability of a marketed bioimpedance (BIA) scale for body composition assessment, in children engaged in an educational football project (FIFA 11 for Health). One-hundred and twenty-seven children (70 boys and 57 girls; age 10.7±0.5 years, body mass 41.2±9.0 kg, Body mass index 18.5±3.3 kg·m^-2^ and stature 149±7 cm) were evaluated for total body mass, lean body mass, muscle mass, using BIA (InBody 270, Biospace, California, USA) and dual-energy X-ray absorptiometry (DEXA, Lunar Prodigy, GE Medical Systems, Madison, Wisconsin, USA), at baseline conditions. Data analyses were carried out separately for girls and boys. Nearly perfect associations (r = 0.97−0.99) and excellent absolute (TEM = 0.04−1.9%) and relative (ICC = 0.98−1.00) inter-device reliability were found between DEXA and BIA variables. Fat and lean body mass bias (p < .0001) were practically relevant both for the boys (2.56 and 11.22 kg, respectively) and the girls (2.33 and 10.49 kg, respectively). Muscle mass and body fat were underestimated and overestimated, respectively, for the boys and girls. InBody 270 is a valid BIA system for estimating body composition with an excellent inter-device relative and absolute reliability. However, the remarkable measurements bias of BIA fat and muscle mass values discourage its use for clinical prescription. The BIA body composition biases were sex dependent.

## Introduction

Body composition has been reported to be associated with health and affected by exercise and diet [1–9]. Specifically, excessive body fat and low muscle mass are linked to cardiovascular and metabolic diseases [2, 4, 10]. Skeletal muscle mass plays an important role in locomotion, metabolism and impacts on other organs due to its secretory function, being an indicator of overall health across the lifecycle [11]. In childhood and adolescence, low muscle mass and strength levels are associated with an elevated risk for cardiometabolic diseases and impairments in neurodevelopment and in bone parameters, increasing the risk of osteoporosis at old age [2, 10]. Moreover, in youth, high adiposity levels and especially abdominal obesity, have been associated with risk factors for cardiovascular disease and metabolic syndrome [12–14].

Assessing and tracking muscle and fat mass throughout children’s development is, therefore, essential to identify relevant health outcomes and functional status, to establish nutritional recommendations, and also to evaluate the effectiveness of nutrition and training interventions on body compositum, aiming to promote health and to prevent diseases [15]. Thus, valid and accurate body composition estimation techniques and devices are needed.

A number of tools and procedures that differentiate for their accuracy and accessibility are currently available to assess body composition [16, 17]. Dual energy X-ray absorptiometry (DEXA) is currently the accepted gold standard for body composition assessment, providing accurate measures of regional body fat and lean body mass [1, 17–21]. The advantage of the DEXA method include observer independence, limited patients’ cooperative demands, high measurement reproducibility. The DEXA body composition measurements have been reported to be affected by scanner and software versions suggesting caution for between studies comparisons [17]. Reduced validity of DEXA variables has been reported only for extreme body composition conditions (i.e. in extremely lean and obese individuals) [17]. However, device costs, operational procedures and logistic issues discourage the use of DEXA in large-scale applied studies [22]. Bioelectrical impedance analysis (BIA) has been proposed as a safe, noninvasive, simple, portable, short-duration, low-cost and feasible alternative to DEXA providing body composition reports that may result acceptable for accuracy and reliability [21–27]. Utilizing the conductive properties of human tissues, the BIA methods is prone to hydration status (extracellular water) [17]. Furthermore, the prediction equation enabling body composition calculations are population specific and providing bias of practical relevance if accurate evaluation is the aim [17].

Originally, body composition assessment via BIA used devices that required subjects to lay down and to be wired to the analyzing unit [21, 23, 27]. Advancements in BIA technology enabled the use of systems requiring subjects standing bare feet on a scale for few seconds. The limited cost and operational ease of BIA scales promoted their use when a large number of subjects needs to be assessed for body composition, especially in field studies [16, 22, 24]. The associated reduced testing time and immediate body composition report provided by BIA scales promoted their success in the fitness industry and clinical set-up [16, 22].

The BIA scales systems assess body composition using proprietary patented algorithms to report the values of interest, mainly total body fat and lean tissues mass (i.e., muscle, bones and flesh) [22]. The published data reported mainly robust association between BIA and studies considered gold standards, with between variables bias being device and population specific [16, 23–27]. However, difference in statistical approach to quantify measurement bias provided inconsistent results [16, 23–26]. The interest over body composition and the practical accessibility of BIA protocols promoted a plethora of marketed scale systems warranting independent validity and reliability research. The reported effects of age, sex, training and health status variability on BIA derived body composition variables promotes the importance of pre-intervention validity and measurement consistency (reliability) of the considered systems [16, 17, 23–26].

The aim of this study was to examine the validity and reliability of a marketed BIA scale for body composition assessment in children participating in an educational football project. In this study were hypothesized sex differences in body composition accuracy operated by BIA device [28, 29].

## Methods

### Design

In this study we tested a commercially available BIA scale marketed as InBody 270 (Biospace, California, USA) for validity and reliability. The InBody 270 multi-frequency system provides body composition data in the form of body fat (BF), body mass (BM) and muscle mass (MM). The BIA derived body composition data were plotted against DEXA (Lunar Prodigy, GE Medical Systems, Madison, Wisconsin, USA) scan data for criterion validity and measurement agreement. Relative and absolute InBody 270 inter-device reliability were assessed with subjects replicating measurements on two devices. Only absolute values of BF were considered, since relative proxies (e.g., % body fat) have been reported to induce fallacious results [30].

Accuracy of BIA devices have been reported to be sex and age-dependent [28]. The accuracy and reliability of the devices were examined considering girls and boys groups separately to avoid spurious correlations [28].

This study was carried out before the implementation of a football-based health-educational program for school children (11 for Health) [31]. The 11 for Health programme aims to use football as an educational vehicle to instil healthy habits in the new generations across the world. Body composition assessment was considered as a 11 for Health outcome to partly evaluate the effectiveness of this programme in children [31]. Validity and reliability of the InBody 270 system was assessed to consider its use in the 11 for Health program.

### Participants

One-hundred and twenty-seven (n = 127, age 10.7±0.5 years, body mass 41.2±9.0 kg, body mass index 18.5±3.3 kg·m^-2^ and stature 149±7 cm) male (n = 70, age 10.8±0.6 years, body mass 42.0±10.1 kg, body mass index 18.6±3.8 kg·m^-2^ and stature 149±8 cm) and female (n = 57, age 10.7±0.5 years, body mass 40.9±7.6 kg, body mass index 18.4±2.6 kg·m^-2^ and stature 149±6 cm) pupils from the Funen region in Denmark volunteered to participate in this study. At the time of this investigation, the children attended the 4 schools from Funen that accepted the invitation to participate in the 11 for Health project (11fH), where all schools in Denmark are invited by e-mail. They signed up for 11fH before June 2019 and was invited for the present study in June by an additional e-mail. They accepted this invitation also in June 2019. The schools came from one big city and three small cities and are considered as representable for all areas in Denmark, except the city centre of the capital. The study was conducted from august to September 2019 at the University of Southern Denmark in Odense. Children and parents/guardians gave their written and informed consents after full explanation of the aims, risks, benefits and procedures associated with the study before its beginning of the study and were made aware that they could withdraw from the study at any time without further notice. The local ethics committee (ethic committee of Southern Denmark) approved this study design and procedures before the commencement of the study.

### Procedures

Children’s body composition was evaluated during a single laboratory visit before the commencement of the FIFA 11 for Health intervention. Measurements were carried out between 8–10 a.m. in each testing occasion. Body composition of all children was measured using two different InBody 270 multifrequency analysers to assess inter-device reliability (InBody 270, Biospace, California, USA). The two BIA assessments were performed one after the others with minimal rest between the trials. Immediately after, a whole body DEXA scan was performed (Lunar Prodigy Advance, GE Medical Systems, Madison, Wisconsin, USA) with data collected using Encore software version 15 (Encore, Madison, WI, USA). The scanner was calibrated from the beginning of all test days. Tests sequence was standardized to avoid changes in body water distribution during the considered procedures [28].

Children were weighted in light clothing and height was measured without footwear with a tallimeter (Tanita, Tanita UK Ltd, Middlesex, UK) before body composition assessments. DEXA considered variables were:

- Total body fat (DEXA^fat^, kg);
- Lean body mass (DEXA^LBM^, kg);
- Body mass (DEXA^BM^, kg).

The DEXA values were compared with the following InBody 270 corresponding variables:

- Total body fat (InBody ^fat^, kg);
- Muscle mass (InBody ^MM^, kg);
- Body mass (InBody ^BM^, kg).

Children were told to drink water *ad libitum* before but not between the measurements in order to standardize their hydration status [17, 28].

### Statistical analyses

All the reported data are expressed as means ± standard deviations and 95% confidence intervals (95% CI), unless stated otherwise. The Shapiro-Wilk W-test was used to verify normality assumption. Measurement agreement was evaluated with equivalence testing [32–34]. The two one sided t-test (TOST) approach was used considering InBody and DEXA variables as surrogate and criterion measure, respectively [32, 34]. Equivalence interval was assumed as ±1 kg, according to minimum measurement difference values reported in healthy men and women in measurement agreement studies [23].

Pearson correlation (r) and regression equations were used to assess the associations between variables. The magnitude of the reported effects was described using the Hopkins et al, (Hopkins et al, 2009a) criteria as follows: trivial, r<0.1; small, 0.1<r<0.3; moderate, 0.3<r<0.5; large, 0.5<r<0.7; very large, 0.7<r<0.9; nearly perfect, r>0.9; and perfect, r = 1. Estimation error was reported as typical error of estimation (TEE) according to the procedures proposed by Hopkins et al, (Hopkins et al, 2009b). Relative reliability was assessed using the intraclass correlation coefficient (ICC 2,1) with 95% CI (Weir 2005; Hopkins 2000). According to Fleiss (Fleiss 2011), ICC values of 0.75–1.00 were considered as excellent, 0.41–0.74, good and 0.00–0.40 poor. Significance was set at 5% (p<0.05) for all calculations. Statistical analyses were performed using R (version 3.5.1, R Foundation for Statistical Computing).

## Results

### Descriptive statistics

The DEXA (n = 124) variables, DEXA^fat^, DEXA^LBM^ and DEXA^BM^ values were 12.0±5.8 kg (11.0–13.1), 27.8±4.3 kg (27.1–28.6), and 41.3±9.1 kg (39.7–43.0), respectively. Values for InBody^fat^, InBody^MM^ and InBody^BM^ variables were 9.6±5.6 kg (8.63–10.65), 16.8±2.9 (16.3–17.3) and 41.3±9.1 kg (39.7–43.0), respectively.

### Inter-device reliability and criterion validity

InBody (n = 88) values for inter-device relative (ICC) and absolute (TEM) reliability were almost perfect and trivial, respectively (Table 1).

**Table 1:** Reliability data of InBody 270 variables (n = 88).

Variables	Fat	Body Mass (kg)	Muscle Mass (kg)
Mean 1	24.2±6.5	41.1±8.8	16.8±2.9
Mean 2	24.4±6.5	41.1±8.8	16.7±2.9
Difference	0.1±0.6	-0.0±0.1	-0.1±0.6
95% CI	-0.01–0.25	-0.01; -0.03	-0.24–0.03
ICC	0.99	1.00	0.98
95% CI	0.98–0.99	1.00–1.00	0.96–0.98
TEM as CV%	0.04	0.1	1.9
95% CI	0.03–0.06	0.1–0.1	1.4–2.8

Fat% = Percentage of body fat; ICC = Intra class correlation coefficient; 95% CI = 95% confidence interval; TEM as CV = Typical Error of the Measurement reported as Coefficient of Variation.

### Measurements agreement

**Fig 1:**
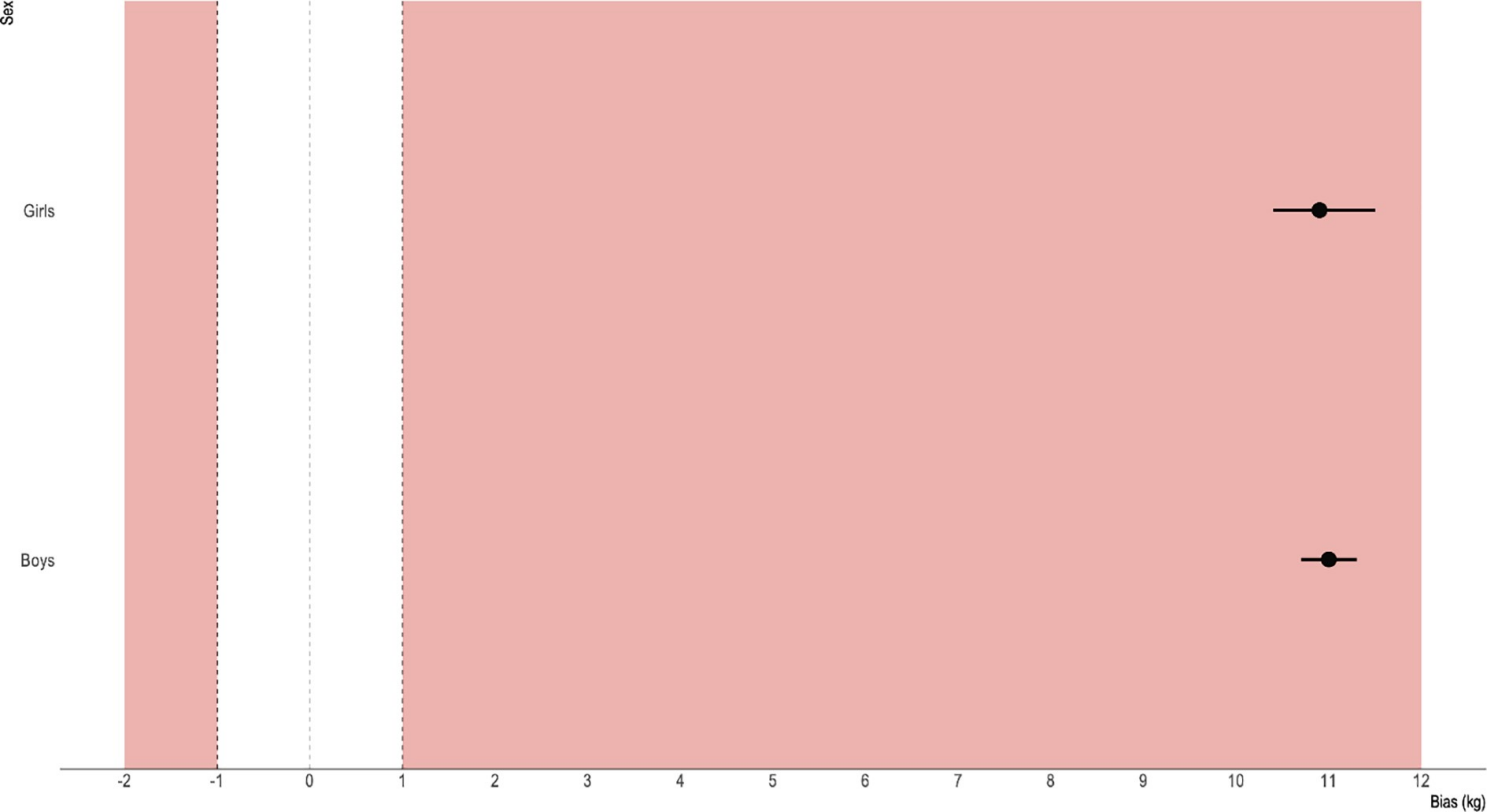


**Fig 2:**
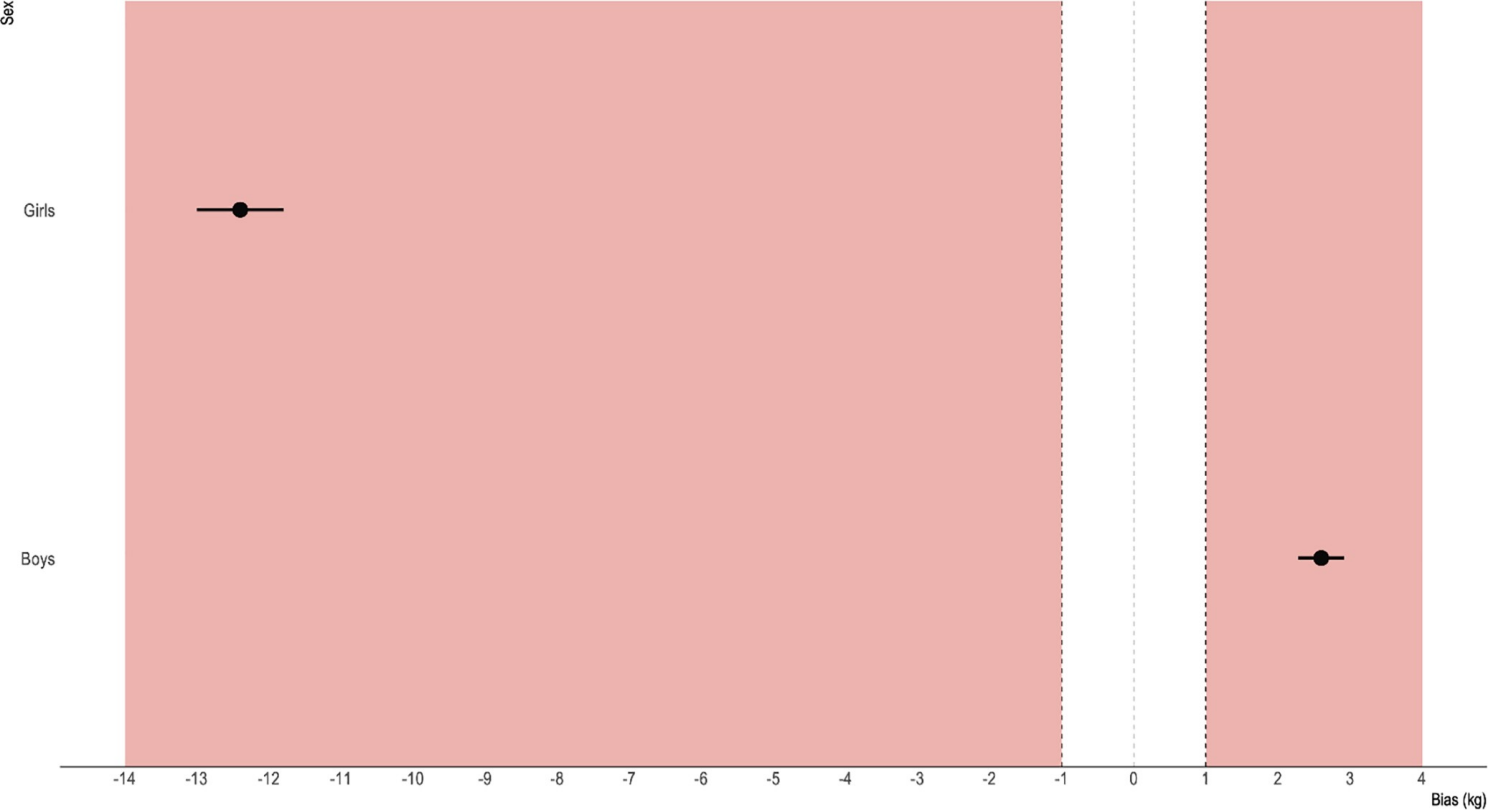


For the boys (n = 69), DEXA and InBody LBM were 27.7±3.9 and 16.7±2.7 kg, respectively. The corresponding values for the girls (n = 54) were 27.3±4.3 and 16.3±2.7 kg. The fat mass values for the boys were 10.8±5.1 and 8.3±4.6 kg for DEXA and InBody, respectively. The girls’ FM values for DEXA and InBody were 12.1±4.4 and 24.5±5.5 kg, respectively. The measurement bias for the boys corresponded to 11.0 (10.7–11.3) kg and 2.6 (2.3–2.9) kg for FFM and FM, respectively. The FFM and FM measurement bias for the girls was 10.9 (10.7–11.3) kg and -12.4 (-13.0− -11.8) kg, respectively. The above between-variables measurement biases were significant (p<0.0001, Tables 2 and 3). Difference plots across variables are reported in Figs 1 and 2.

**Table 2:** Boys’ measurement agreement values (kg) for DEXA vs InBody variables comparison (n = 69).

Variables	Fat	Body Mass	LBM vs. MM
**Difference**	2.56	0.35	11.22
**SD**	1.32	2.40	1.51
**95% CI**	2.24–2.88	-0.22–0.93	10.86–11.58
**P value**	<0.0001	0.23	<0.0001
**Upper limit**	5.15	5.05	14.19
**95% CI**	4.60–5.69	4.06–6.04	13.56–14.81
**Lower limit**	-0.03	-4.34	8.26
**95% CI**	-0.57–0.52	-5.33–3.35	7.63–8.88

**Table 3:** Girls measurements agreement values (kg) of DEXA vs. InBody variables comparison (n = 55).

Variables	Fat	Body Mass	LBM vs. MM
**Difference**	2.33	-0.01	10.49
**SD**	1.07	0.18	3.64
**95% CI**	2.04–2.62	-0.06–0.04	9.51–11.48
**P value**	<0.0001	0.5848	<0.0001
**Upper limit**	4.4190	0.3474	17.62
**95% CI**	3.93–4.91	0.26–0.43	15.93–19.31
**Lower limit**	0.24	-0.38	3.36
**95% CI**	-0.25–0.74	-0.46–0.29	1.67–5.05

### Associations between variables

Associations between DEXA and InBody variables were nearly perfect (Table 4) in girls and boys groups, but the correlation between DEXA^LBM^ and InBody ^MM^ in the girls group was large.

**Table 4:** Associations between DEXA vs. InBody variables in the boys and girls group.

	Variables	Fat	Body Mass	LBM vs. MM
**Boys** (n = 69)	**r**	0.98	0.97	0.98
	**95% CI**	0.97–0.99	0.96–0.98	0.97–0.99
	**P value**	<0.0001	<0.0001	<0.0001
**Girls** (n = 55)	**r**	0.97	0.99	0.67
	**95% CI**	0.95–0.98	0.99–1.00	0.50–0.80
	**P value**	<0.0001	<0.0001	<0.0001

## Discussion

This study results showed BIA system to possess excellent group (i.e., boys and girls) relative and absolute reliability. Nearly perfect associations were found between DEXA and InBody variables across sex, providing evidence for criterion validity. However, a large measurement bias between DEXA and InBody were reported showing descriptive evidence of BIA device poor accuracy in profiling body composition in children of different sex. In light of this study findings, when interpreting muscle mass and body fat values using the InBody 270 system, caution is suggested. Given the reliability of the InBody 270 (i.e., measurement precision) longitudinal studies examining the sensitiveness of the considered device in detecting training-induced intra- and inter-subjects body composition variations are warranted.

Obesity pandemic increased the interest in body composition assessment in children [6, 35]. Diet, exercise and education for healthy lifestyles have been proposed as effective strategies to promote positive changes in body composition [8, 9]. The FIFA 11 for Health program was devised with the aim to combine exercise benefits and healthy lifestyle education using football in youngsters [31, 36]. Variability in intervention outcomes and clinical practise suggest the recurrent use of sustainable body composition methods [24], with the aim to control and regulate the training process in order to allow for individual adjustments in training doses and daily healthy lifestyle behaviour.

The BIA assessment sustainability, both economical and procedural, has promoted a number of validity and reliability independent studies of the several marketed systems in selected populations [16]. Patient’s age, sex, hydration status, adiposity and muscularity have been reported to influence body composition values warranting BIA systems validation studies [26].

Maturation stages providing for a given chronological age, variability in biological age may potentially affect BIA values in children [16, 23, 28].

A recent study, carried out in 7-12-year-old Taiwanese children, tested a number of BIA systems against DEXA scanner for validity, reporting very accurate muscle mass values [37]. Interestingly, BIA systems were precise though bringing clinically significant errors in estimating body composition variables in children (i.e., fat mass and relative body fat underestimation) [37]. Studying 10–17year-old boys and girls, InBody devices produced significantly different percentages of body fat when using underwater weight as reference in girls [28]. Mclester et al. [23] tested InBody BIA devices for validity and reliability in the general population reporting systematic bias. Specifically, the cited authors found body fat percentage and fat mass underestimation with proportional bias present for fat mass in women and free fat mass in men [23].

Researches that examined BIA accuracy, found 7% lower body fat percentage values than reported DEXA reference values [24]. The values locating in the range of difference (-16.9–7.7%) usually reported for BIA vs. reference (i.e., DEXA and underwater weighting) measures [16].

The published body of evidence draw measurement agreement inferences mostly using the Bland and Altman approach [16, 21–24, 27, 37]. The considered limits of agreement provide unscaled absolute values that enable the visual (i.e., Bland and Altman difference vs. average plot) and quantitative (i.e., measurement bias) inspection of the difference between the reference and surrogate measure [38]. However, the interpretation of the clinical relevance of the resulting differences requires a priori familiarity with the variables considered [37].

In this study, the equivalence testing method to assess measurement agreement was used for the first time in BIA validation studies [32–34]. This method was implemented using previously published reference data to set equivalence intervals. For this study variables, we considered the minimum difference of ±1 kg as a priori interval of equivalence [23, 34]. Additionally, only absolute variables values were considered to avoid flawed data [30].

Using these novel methodological approaches, we found remarkable data bias for the variables here considered. This despite the nearly perfect (i.e., 0.97–0.99) and excellent (i.e., 0.98–0.99) DEXA vs. BIA associations and reliability. The reported associations’ magnitude is in line with previous studies using similar research designs and addressing children and adults [16, 23, 24, 26, 37]. In light of this findings, InBody reveals as a valid method (i.e., criterion validity). However, the reported bias discourages diagnostic accuracy, suggesting, at best, data precision. It could be speculated that InBody 270 may be used to track individual longitudinal variations in body composition, but future studies are warranted.

In this study, muscle mass values bias was related to children sex, with BIA providing a huge overestimation and practical important underestimation in girls and boys, respectively. Interestingly, the fat mass bias was aligned in girls and boys, showing a huge method underestimation. Evidence for sex differences in BIA body composition estimations were reported for 7-12-year-old Taiwanese children [37]. Differently from this study, the cited paper found the reported BIA device (i.e., InBody 230) to underestimate and overestimate relative body fat in girls and boys, respectively. No sex differences were reported in muscle mass accuracy [37]. The between studies difference in body composition estimate values may be ascribed to the diverse BIA devices considered, and the use of different analytical methods for assessing differences and reporting data.

These findings are similar with reliability and validity values of BIA devices from other manufactures in children and adult [16, 26, 29]. The differences depend on sex and body weight status, highlighting the importance of considering the homogeneity of the study group, when interpreting the BIA analysis results.

Whereas the strengths of this study is the novel and highly relevant target group, as well as the immediate followed measures (Inbody and DEXA, a limitation could be the risk, that the children did not follow the instructions to fast at least one hour prior to the measures.

Also, the physiological day-to-day variations must be taken into account. This is highlighted in a study by Andersen et al. [39] evaluating the precision of a BIA device (Xitron 4200) different from that analysed in this study. The precision was 0.5–2.4% in children aged 6–14 years. Within-day variation was 1.1–2.8% and between-day variation was 2.4–5.7%, indicating that day-to-day variation in hydration affects the measures.

## Conclusions

Together, these results support the criterion validity and inter-device reliability of Inbody 270 device in children of different sex. However, the magnitude of the reported bias strongly discourage the use of InBody 270 to provide accurate clinical information of body fat and muscle mass in children. The measurement bias showed to be related with children sex. Specifically, muscle mass was heavily overestimated in girls. Given the reported satisfactory relative and absolute inter-device reliability, the InBody 270 device appear useful in longitudinal studies to provide precise body composition data collection. However, longitudinal studies examining the sensitiveness of BIA in detecting training-induced intra and inter-subjects’ body composition variations are warranted.

## Supporting information

S1 File (XLSX)

## References

[1] Thibault R, Pichard C. The evaluation of body composition: a useful tool for clinical practice. Ann Nutr Metab. 2012;60(1):6–16. 10.1159/000334879 22179189

[2] Srikanthan P, Horwich TB, Tseng CH. Relation of Muscle Mass and Fat Mass to Cardiovascular Disease Mortality. Am J Cardiol. 2016;117(8):1355–60. 10.1016/j.amjcard.2016.01.033 26949037

[3] Landry BW, Driscoll SW. Physical activity in children and adolescents. PM & R: the journal of injury, function, and rehabilitation. 2012;4(11):826–32. 10.1016/j.pmrj.2012.09.585 23174545

[4] Kim JH, Park YS. Low muscle mass is associated with metabolic syndrome in Korean adolescents: the Korea National Health and Nutrition Examination Survey 2009–2011. Nutr Res. 2016;36(12):1423–8. 10.1016/j.nutres.2016.09.013 27884414

[5] Hens W, Vissers D, Hansen D, Peeters S, Gielen J, Van Gaal L, et al. The effect of diet or exercise on ectopic adiposity in children and adolescents with obesity: a systematic review and meta-analysis. Obesity reviews: an official journal of the International Association for the Study of Obesity. 2017;18(11):1310–22. 10.1111/obr.12577 28913977

[6] Gutin B, Islam S, Manos T, Cucuzzo N, Smith C, Stachura ME. Relation of percentage of body fat and maximal aerobic capacity to risk factors for atherosclerosis and diabetes in black and white seven- to eleven-year-old children. J Pediatr. 1994;125(6 Pt 1):847–52. 10.1016/s0022-3476(05)81997-3 7996354

[7] Brown T, Moore TH, Hooper L, Gao Y, Zayegh A, Ijaz S, et al. Interventions for preventing obesity in children. The Cochrane database of systematic reviews. 2019;7(7):Cd001871. 10.1002/14651858.CD001871.pub4 31332776 PMC6646867

[8] Ouchi N, Parker JL, Lugus JJ, Walsh K. Adipokines in inflammation and metabolic disease. Nat Rev Immunol. 2011;11(2):85–97. 10.1038/nri2921 21252989 PMC3518031

[9] Zeng Q, Dong SY, Sun XN, Xie J, Cui Y. Percent body fat is a better predictor of cardiovascular risk factors than body mass index. Braz J Med Biol Res. 2012;45(7):591–600. 10.1590/s0100-879x2012007500059 22510779 PMC3854278

[10] Orsso CE, Tibaes JRB, Oliveira CLP, Rubin DA, Field CJ, Heymsfield SB, et al. Low muscle mass and strength in pediatrics patients: Why should we care? Clin Nutr. 2019;38(5):2002–15. 10.1016/j.clnu.2019.04.012 31031136

[11] Pedersen BK, Febbraio MA. Muscles, exercise and obesity: skeletal muscle as a secretory organ. Nat Rev Endocrinol. 2012;8(8):457–65. 10.1038/nrendo.2012.49 22473333

[12] Reed KE, Warburton DE, Macdonald HM, Naylor PJ, McKay HA. Action Schools! BC: a school-based physical activity intervention designed to decrease cardiovascular disease risk factors in children. Prev Med. 2008;46(6):525–31. 10.1016/j.ypmed.2008.02.020 18377970

[13] Tjønna AE, Stølen TO, Bye A, Volden M, Slørdahl SA, Odegård R, et al. Aerobic interval training reduces cardiovascular risk factors more than a multitreatment approach in overweight adolescents. Clin Sci (Lond). 2009;116(4):317–26. 10.1042/CS20080249 18673303

[14] Williams DP, Going SB, Lohman TG, Harsha DW, Srinivasan SR, Webber LS, et al. Body fatness and risk for elevated blood pressure, total cholesterol, and serum lipoprotein ratios in children and adolescents. Am J Public Health. 1992;82(3):358–63. 10.2105/ajph.82.3.358 1536350 PMC1694353

[15] Madden AM, Smith S. Body composition and morphological assessment of nutritional status in adults: a review of anthropometric variables. J Hum Nutr Diet. 2016;29(1):7–25. 10.1111/jhn.12278 25420774

[16] Talma H, Chinapaw MJ, Bakker B, HiraSing RA, Terwee CB, Altenburg TM. Bioelectrical impedance analysis to estimate body composition in children and adolescents: a systematic review and evidence appraisal of validity, responsiveness, reliability and measurement error. Obesity reviews: an official journal of the International Association for the Study of Obesity. 2013;14(11):895–905. 10.1111/obr.12061 23848977

[17] Fosbøl M, Zerahn B. Contemporary methods of body composition measurement. Clin Physiol Funct Imaging. 2015;35(2):81–97. 10.1111/cpf.12152 24735332

[18] Salamat MR, Shanei A, Khoshhali M, Salamat AH, Siavash M, Asgari M. Use of conventional regional DXA scans for estimating whole body composition. Arch Iran Med. 2014;17(10):674–8. 25305766

[19] Hangartner TN, Warner S, Braillon P, Jankowski L, Shepherd J. The Official Positions of the International Society for Clinical Densitometry: acquisition of dual-energy X-ray absorptiometry body composition and considerations regarding analysis and repeatability of measures. J Clin Densitom. 2013;16(4):520–36. 10.1016/j.jocd.2013.08.007 24183641

[20] Fowke JH, Matthews CE. PSA and body composition by dual X-ray absorptiometry (DXA) in NHANES. Prostate. 2010;70(2):120–5. 10.1002/pros.21039 19739130 PMC2798924

[21] Buckinx F, Reginster JY, Dardenne N, Croisiser JL, Kaux JF, Beaudart C, et al. Concordance between muscle mass assessed by bioelectrical impedance analysis and by dual energy X-ray absorptiometry: a cross-sectional study. BMC Musculoskelet Disord. 2015;16:60. 10.1186/s12891-015-0510-9 25887598 PMC4369090

[22] Aandstad A, Holtberget K, Hageberg R, Holme I, Anderssen SA. Validity and reliability of bioelectrical impedance analysis and skinfold thickness in predicting body fat in military personnel. Military medicine. 2014;179(2):208–17. 10.7205/MILMED-D-12-00545 24491619

[23] McLester CN, Nickerson BS, Kliszczewicz BM, McLester JR. Reliability and Agreement of Various InBody Body Composition Analyzers as Compared to Dual-Energy X-Ray Absorptiometry in Healthy Men and Women. J Clin Densitom. 2018. 10.1016/j.jocd.2018.10.008 30472111

[24] Kabiri LS, Hernandez DC, Mitchell K. Reliability, Validity, and Diagnostic Value of a Pediatric Bioelectrical Impedance Analysis Scale. Childhood obesity (Print). 2015;11(5):650–5. 26332367 10.1089/chi.2014.0156

[25] Leahy S, O’Neill C, Sohun R, Jakeman P. A comparison of dual energy X-ray absorptiometry and bioelectrical impedance analysis to measure total and segmental body composition in healthy young adults. Eur J Appl Physiol. 2012;112(2):589–95. 10.1007/s00421-011-2010-4 21614505

[26] Völgyi E, Tylavsky FA, Lyytikäinen A, Suominen H, Alén M, Cheng S. Assessing body composition with DXA and bioimpedance: effects of obesity, physical activity, and age. Obesity (Silver Spring, Md). 2008;16(3):700–5. 10.1038/oby.2007.94 18239555

[27] Jackson AS, Pollock ML, Graves JE, Mahar MT. Reliability and validity of bioelectrical impedance in determining body composition. Journal of applied physiology. 1988;64(2):529–34. 10.1152/jappl.1988.64.2.529 3372410

[28] Jensky-Squires NE, Dieli-Conwright CM, Rossuello A, Erceg DN, McCauley S, Schroeder ET. Validity and reliability of body composition analysers in children and adults. Br J Nutr. 2008;100(4):859–65. 10.1017/S0007114508925460 18346304

[29] Hosking J, Metcalf BS, Jeffery AN, Voss LD, Wilkin TJ. Validation of foot-to-foot bioelectrical impedance analysis with dual-energy X-ray absorptiometry in the assessment of body composition in young children: the EarlyBird cohort. Br J Nutr. 2006;96(6):1163–8. 10.1017/bjn20061960 17181893

[30] Cole TJ, Fewtrell MS, Prentice A. The fallacy of using percentage body fat as a measure of adiposity. Am J Clin Nutr. 2008;87(6):1959; author reply -60. 10.1093/ajcn/87.6.1959 18541591

[31] Skoradal MB, Purkhús E, Steinholm H, Olsen MH, Ørntoft C, Larsen MN, et al. "FIFA 11 for Health" for Europe in the Faroe Islands: Effects on health markers and physical fitness in 10- to 12-year-old schoolchildren. Scand J Med Sci Sports. 2018;28 Suppl 1:8–17. 10.1111/sms.13209 29882318

[32] Dixon PM, Saint-Maurice PF, Kim Y, Hibbing P, Bai Y, Welk GJ. A Primer on the Use of Equivalence Testing for Evaluating Measurement Agreement. Med Sci Sports Exerc. 2018;50(4):837–45. 10.1249/MSS.0000000000001481 29135817 PMC5856600

[33] Siebert M, Ellenberger D. Validation of automatic passenger counting: introducing the t-test-induced equivalence test. Transportation. 2019.

[34] Lakens D. Equivalence Tests: A Practical Primer for t Tests, Correlations, and Meta-Analyses. Soc Psychol Personal Sci. 2017;8(4):355–62. 10.1177/1948550617697177 28736600 PMC5502906

[35] Zimmet P, Alberti KG, Kaufman F, Tajima N, Silink M, Arslanian S, et al. The metabolic syndrome in children and adolescents—an IDF consensus report. Pediatr Diabetes. 2007;8(5):299–306. 10.1111/j.1399-5448.2007.00271.x 17850473

[36] Lind RR, Geertsen SS, Ørntoft C, Madsen M, Larsen MN, Dvorak J, et al. Improved cognitive performance in preadolescent Danish children after the school-based physical activity programme "FIFA 11 for Health" for Europe—A cluster-randomised controlled trial. Eur J Sport Sci. 2018;18(1):130–9. 10.1080/17461391.2017.1394369 29161988

[37] Lee LW, Liao YS, Lu HK, Hsiao PL, Chen YY, Chi CC, et al. Validation of two portable bioelectrical impedance analyses for the assessment of body composition in school age children. PLoS One. 2017;12(2):e0171568. 10.1371/journal.pone.0171568 28158304 PMC5291432

[38] Bland JM, Altman DG. Statistical methods for assessing agreement between two methods of clinical measurement. Lancet. 1986;1(8476):307–10. 2868172

[39] Andersen TB, Jødal L, Arveschoug A, Eskild-Jensen A, Frøkiær J & Brøchner-Mortensen J. Precision and within-and between-day variation of bioimpedance parameters in children aged 2–14 years. *Clinical nutrition*, 2011;30(3), 326–331. 10.1016/j.clnu.2010.10.005 21074302

